# Association of pharmaceutical care barriers and role ambiguity and role conflict of clinical pharmacists

**DOI:** 10.3389/fphar.2023.1103255

**Published:** 2023-05-09

**Authors:** Qingran Sun, Chuchuan Wan, Zhaoqi Xu, Yuankai Huang, Xiaoyu Xi

**Affiliations:** National Medical Products Administration Key Laboratory for Drug Regulatory Innovation and Evaluation, Nanjing, Jiangsu, China

**Keywords:** role ambiguity, role conflict, pharmaceutical care, barriers, clinical pharmacists, China

## Abstract

**Objectives:** This study aimed to understand current status of pharmaceutical care barriers and explore the impact of them on the role ambiguity and role conflict of clinical pharmacists in secondary and tertiary hospitals in mainland China.

**Methods:** The Chinese version of Role Conflict and Role Ambiguity Scale was used to measure clinical pharmacists’ role ambiguity and role conflict. A questionnaire for clinical pharmacists’ pharmaceutical care barriers was established to determine whether clinical pharmacists encounter barriers. Multiple linear regression model was used to analyze the influence of various pharmaceutical care barriers on the role ambiguity and role conflict of clinical pharmacists.

**Results:** 1,300 clinical pharmacists from 31 provinces were eventually included. The results revealed that commonly perceived barriers to pharmaceutical care by clinical pharmacists include the lack of financial compensation and dedicated time for pharmaceutical care. Barriers such as clinical pharmacists’ unawareness of the importance of pharmaceutical care increase the degree of clinical pharmacists’ role conflict. And the lack of financial compensation for pharmaceutical care decreases the degree of role ambiguity, while barriers such as the lack of dedicated time for pharmaceutical care, the failure to standardize the service procedures and contents of related documents in healthcare institutions increase the degree of role ambiguity.

**Conclusion:** Increased focus on enhancing financial compensation, responsibility cognition, education and training, and greater consideration of institutional factors could help clinical pharmacists better manage their work environments and provide higher-quality pharmaceutical care.

## 1 Introduction

As an expert in medicines of the multidisciplinary team, pharmacists play a key role in ensuring rational drug use (Avery et al., 2012). Studies have reported the positive impact of the participation of the clinical pharmacists in solving drug-related issues, reducing readmission rates, preventing adverse drug events, improving patients’ adherence, and alleviating medical care costs (Vulaj et al., 2018; Perrot et al., 2019; Hadi et al., 2014; Foppa et al., 2016). The pharmaceutical care was defined as “the contribution made by pharmacists to optimize the use of drugs and improve the health of patients” (Allemann et al., 2014), which has been established in many countries. As early as 2008, a European survey showed that pharmaceutical care would bring good outcomes in terms of patients’ medicine therapy. Over the years, increasing attention has been paid to pharmaceutical care for improving clinical outcomes and lightening economic burden (Gallagher et al., 2014) and many countries including the US, the UK, Canada have been providing pharmaceutical care (Martin-Calero et al., 2004; Farris et al., 2005). While in China, pharmaceutical care in hospitals is still in the development stage.

The pharmaceutical care is mainly provided in secondary and tertiary hospitals in China. Clinical pharmacists provide pharmaceutical care such as dispensing, training and teaching, checking prescriptions, drug monitoring, making treatment plans, patient education, pharmacy consultation, joining clinical rounds, medication guidance and pharmaceutical monitoring (Guo et al., 2020). A systematic review (Penm et al., 2014a) and several empirical researches (Shao et al., 2017; Xie et al., 2020; Ding et al., 2022) in China reported that pharmaceutical care saved medical costs and positively influenced health outcomes by increasing appropriate use of medication, patient knowledge, quality of life, adherence and reducing patient length of hospitalization. In recent years, the Chinese authorities strongly support the implementation of pharmaceutical care. To further promote and standardize pharmaceutical care, the National Health Commission (NHC) promulgated Opinions on Accelerating the High-quality Development of Pharmaceutical Care(National Health Commission, 2018a) and formulated service specifications for pharmacist-managed clinics, drug use education and pharmaceutical monitoring service in medical institutions (National Health Commission, 2021).

However, in many developing countries including China, the implementation of pharmaceutical care has been suboptimal (Katoue and Ker, 2018; Doloresco and Vermeulen, 2009) and the development of clinical pharmacist care systems is hindered by unclear cognition of clinical pharmacists’ roles (Yu et al., 2016). Role cognition includes two dimensions: role ambiguity and role conflict (Kahn and Rosenthal, 1965). Role conflict was defined as the degree to which expectations of a role of an individual are incompatible or incongruent with the values, abilities, and expertise of the role incumbent. Role ambiguity was as the extent to which an individual is unclear about the expectations of others as well as the degree of uncertainty associated with one’s performance (Kahn and Rosenthal, 1964). Role ambiguity and role conflict may prevent clinical pharmacists from undertaking their responsibilities and achieving desired outcomes of pharmaceutical care (Li et al., 2020b). Understanding role conflict and role ambiguity not only helps clinical pharmacists to understand their responsibilities but also improves the pharmaceutical care practice in health institutions.

Clinical pharmacists’ responsibilities and roles have been appropriately defined in many countries (Seaton et al., 2000; Dawoud et al., 2011). However, the responsibilities of clinical pharmacists are not clear in China (Minister of Health of the People’s Republic of China, 2011). The Regulations on the Administration of Pharmaceutical Affairs in Medical Institutions (Draft for Comment) in 2010 and the subsequent official draft in 2011 have changed the division of responsibilities of clinical pharmacists in China. The lack of regulation, and vague and frequently changing instructions may lead to role ambiguity (House and Rizzo, 1972). In practice, clinical pharmacists in China are always required to participate in clinical pharmacy services and non-clinical work (Li et al., 2018; Dong-ning et al., 2019). Different subjects (e.g., physicians, nurses) and different departments have different requirements for clinical pharmacists (Yuan-kai et al., 2019; Xiao-yu et al., 2018), which may lead to role conflict. Moreover, work mode of clinical pharmacists in China is in transition from a drug orientation (such as drug supply and prescription checking) to a patient orientation (such as pharmaceutical care) (PRC, 2018) and shift in responsibilities may lead to role ambiguity and role conflict (Taylor et al., 2020). Therefore, clinical pharmacists in China may currently experience role ambiguity and role conflict.

Some scholars have studied the role of Chinese clinical pharmacists, however, the research on role ambiguity and role conflict is missing (DS et al., 2017; YY et al., 2017). At present, the subjects of studies on role ambiguity and role conflict mainly focus on nurses, physicians, and community pharmacists (Allison and Simone, 2013; Wenqian et al., 2013; Y et al., 2009). Rieck believed that Australian physicians do not have a clear understanding of the role of community pharmacists and lack trust in the capabilities of community pharmacists, thus resulting in community pharmacists’ role conflict (Allison and Simone, 2013). Guirguis analyzed from an organizational perspective that role stressors such as role conflict, ambiguity and overload can affect pharmacists’ work life. Due to factors such as differences in economic development, health systems and working mode, clinical pharmacists in China are quite different from pharmacists in other countries, therefore, these conclusions should be considered with caution.

Some scholars have increasingly paid attention to barriers in pharmaceutical care which refers to various factors that hinder or negatively impact the work of clinical pharmacists when they provide pharmaceutical care. Barriers to hospital pharmaceutical care encountered by Chinese clinical pharmacists come from external factors (such as lack of administrative support from the government and hospitals, patient distrust, salary, location and rank of hospitals), resources constraints (such as shortage of pharmacy staff, insufficient training of pharmacists, lack of service guidance, insufficient experience and knowledge of pharmacists), and expectations and self-concept of clinical pharmacists (such as unconfident, limited career development, under-appreciated by leaders, physicians and patients), etc., (Penm et al., 2014b; Lounsbery et al., 2009). The role cognition of pharmacists is an important component of pharmaceutical care barriers, which potentially indicate that pharmaceutical care barriers may affect the role ambiguity and role conflict of clinical pharmacists. Moreover, occupational climate conditions are sources of role ambiguity and role conflict (Senatra, 1980; Sanli et al., 2021). As pharmaceutical care barriers are part of pharmacist’s occupational climate conditions, pharmaceutical care barriers may be a potential source of pharmacists’ role ambiguity and role conflict. At present, there is no study that have explored the quantitative relationship between pharmaceutical care barriers and role ambiguity, role conflict of clinical pharmacists in China.

This study intends to comprehensively know about the current situation of pharmaceutical care barriers of clinical pharmacists in secondary and tertiary hospitals in China, investigate whether the pharmaceutical care barriers affect the role conflict and role ambiguity of clinical pharmacists and provide potential solutions to existing problems concerning pharmaceutical care barriers. We expect that the findings would provide Chinese healthcare authorities with advice on the improvement of clinical pharmacist professionalism and removal of the barriers in pharmaceutical care provision. The study could also provide references for other similar developing countries.

## 2 Materials and methods

### 2.1 Setting and study design

A multi-stage sampling strategy was adopted: 1) cities within each province in mainland China (a total of 31 provinces/autonomous regions/municipalities) were divided into three groups according to their *per capita* GDP in 2018.2) At least 2 secondary and 2 tertiary hospitals were selected using convenient sampling in each group based on approval of hospital administrators. 3) At least 2 clinical pharmacists were selected using convenient sampling in each hospital based on their willingness. The inclusion criteria were: 1) worked full-time; 2) available and willing to participate in the survey that would take approximately 30 min; and 3) able to sign the informed consent document. Clinical pharmacists in clerkships and those accepting training were excluded.

**FIGURE 1:**
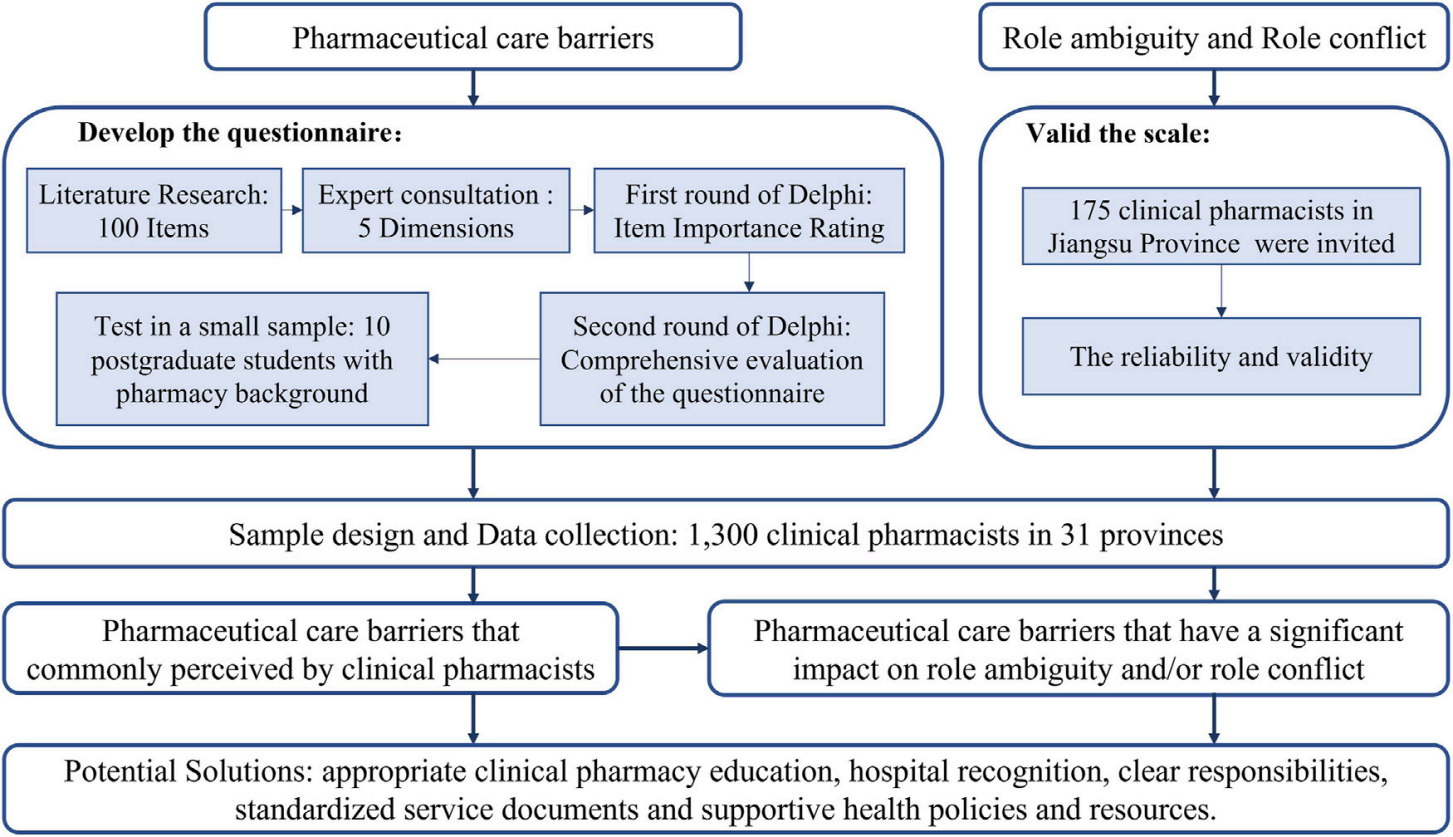
Flowchart of the study.

A minimum sample size of 663 participants was calculated from Raosoft calculator (Raosoft., 2008). The total estimated size is 468,000 (National Health Commission, 2018b) with a 99% confidence level, ±5% margin of error, and 50% response distribution. The flow of this study was shown in Figure 1

### 2.2 Instrument

#### 2.2.1 Role conflict and role ambiguity

Clinical pharmacists’ role ambiguity and role conflict were measured by the Chinese version of Role Conflict and Role Ambiguity Scale (Attachment 1) (Hua et al., 2015). The original scale included 8 items measuring role conflict and 6 items measuring role ambiguity (Rizzo JR and SI, 1970). A 7-point Likert scale was applied, ranging from “completely disagree” (1 point) to “completely agree” (7 points). The items of role conflict are all positively coded, which means the higher the score is, the deepener degree of role conflict is. Conversely, the items of role ambiguity are negatively coded. We conducted pre-research in Jiangsu Province and invited 175 clinical pharmacists to complete the questionnaire. The alpha reliability coefficient method was used to verify the reliability of the questionnaire. The role ambiguity scale has acceptable reliability and validity: Cronbach’s alpha = 0.853, The Kaiser–Meyer–Olkin measurement of sampling adequacy = 0.860; The Barlett’s Test of Sphericity = 576.460, d. f. = 15, *p* = 0.000. The role conflict scale has acceptable reliability and validity: Cronbach’s alpha = 0.779, The Kaiser–Meyer–Olkin measurement of sampling adequacy = 0.779; The Barlett’s Test of Sphericity = 400.967, d. f. = 28, *p* = 0.000.

#### 2.2.2 Pharmaceutical care barriers

The existing tools are not suitable for measuring pharmaceutical care barriers in Chinese hospitals due to poor cross-cultural adaptability (Liang-jiang et al., 2022). Therefore, these barriers were measured by a self-developed questionnaire.

At first, we reviewed literatures (Lounsbery et al., 2009; Scahill et al., 2009; Jorgenson et al., 2014) and found that there was no consensus on the tools for measuring pharmaceutical care barriers in hospital. Then, we identified five dimensions of pharmaceutical care barriers including pharmacist, collaboration, resource, social and financial dimension and allocated 100 items according to the dimensions by literature review and expert consulting. Next, through the first round of consultation using Delphi method, we summarized possible pharmaceutical care barriers in Chinese hospitals. Later, we combined similar barriers and eliminated secondary barriers to form the first draft of the questionnaire. After initial discussion, 15 experts were invited to the second round of consultation to evaluate each part of the questionnaire. Having received their feedback, we modified the first draft to obtain the initial questionnaire. Finally, we randomly selected 10 postgraduate students with pharmacy background to take initial test and verify the readability and understandability of the questionnaire. According to the results, the unclear content was modified and the final questionnaire included 27 questions with a choice of agreement or not. The option of “agreement” indicated that the clinical pharmacist significantly perceived corresponding pharmaceutical care barrier, *vice versa*. More details of the development of pharmaceutical care barriers questionnaire were shown in Supplementary Table S6.

### 2.3 Covariate variables

The study included gender, age, marital status, basic information of children, working years, title and education as control variables. These factors have been shown to have an impact on role ambiguity and role conflict (Sanli et al., 2021; Wells, 2021).

### 2.4 Data collection

A total of 510 undergraduates majoring in pharmacy were recruited as data-collectors. The field survey was conducted during July and August of 2019. With the hospital administrator’s consent, data-collectors entered the pharmacy department and then asked the potential participants for their information to determine whether they met the inclusion criteria., The data-collectors informed potential respondents of the purpose and contents of this survey to ensure the validity and quality of the data. Every participant filled in a structured and pre-coded questionnaire through online survey system after signing the informed consent. The data-collectors were required to assist only if the participants had doubt on how to interpret any question from the questionnaire.

### 2.5 Statistical analysis

The multiple linear regression models were used to analyze the impact of pharmaceutical care barriers on clinical pharmacists’ role ambiguity and role conflict. Multicollinearity was assessed by examining the variance inflation factor (VIF). An independent variable with a VIF value more than 10 should be removed. To evaluate the robustness of the results, only pharmaceutical care barriers were included in the regression. And all covariates were divided into two groups: basic personal and work-related variables. Then, work-related and basic personal variables were included in the sequence. Three models were finally developed. The similarity of three models supported the relative robustness of the final model. Data analysis was performed by using SPSS 26.0.

## 3 Results

A total of 1,300 respondents were included, whose sociodemographic characteristics were shown in Table 1. The average age and working years were 35.65 and 10.04 years, respectively. Respondents’ average score of role ambiguity and role conflict were lower than neutral point of measure at 33.80 and 27.70, respectively.

**TABLE 1:** Demographic characteristics.

The variable name	N/Proportion (%)
Age (Mean, %)	35.65 (7.14)
Working years (Mean, %)	10.04 (7.33)
Role ambiguity (Mean, %)	33.80 (4.97)
Role conflict (Mean, %)	27.70 (7.20)
Gender	
Male	454 (34.9%)
Female	846 (65.1%)
Marital status	
Unmarried	211 (16.2%)
Married	1,081 (83.2%)
Others (divorce, widowhood, etc.)	8 (0.6%)
Whether have children	
Yes	76.1%
No	23.9%
Technical title junior title	451 (34.7%)
Intermediate title	677 (52.1%)
Deputy senior title senior title	147 (11.3%)
	25 (1.9%)
Education background	
Lower than Bachelor’s degree^a^	99 (7.6%)
Bachelor’s degree	821 (63.2%)
Master’s degree	369 (28.4%)
PhD	11 (0.8%)
Qualification	
Nationally-trained specialist clinical pharmacist	383 (29.5%)
Nationally-trained general clinical pharmacist	240 (18.5%)
Provincial-trained specialist clinical pharmacist	298 (22.9%)
Provincial-trained general pharmacist	264 (20.3%)
Untrained clinical pharmacist^b^	295 (22.7%)
Profession	
Anti-Infective Program	330 (25.4%)
Cardiovascular Medicine	211 (16.2%)
Respiratory Medicine	219 (16.8%)
Gastroenterology	207 (15.9%)
Nephrology	76 (5.8%)
Antitumor Medicine	100 (7.7%)
Organ Transplantation	18 (1.4%)
ICU	69 (5.3%)
Endocrinology	112 (8.6%)
Neurology	100 (7.7%)
Other	303 (23.3%)
Participation in training and qualification acquisition	
Certificate of completion of clinical pharmacist training from the Ministry of Health	602 (46.3%)
Clinical pharmacist advanced training certificate	432 (33.2%)
Clinical pharmacist training teacher certificate of the Health Planning Commission	278 (21.4%)
Go abroad to participate in relevant clinical pharmacist training	32 (2.5%)
Other training	231 (17.8%)

^a^ In China, clinical pharmacists have been required to have a bachelor’s degree or higher since 2011, while in-service clinical pharmacists with substandard education were allowed to continue practicing after standardized training. For this historical reason, a small number of clinical pharmacists with substandard educational backgrounds have rich clinical experience and are capable of completing clinical pharmacy work.

^b^ “Untrained clinical pharmacist” means no training experience in any of the above four.

PhD = doctor of philosophy, ICU, intensive care unit.

Table 2 showed respondents’ perceptions of pharmaceutical care barriers. Most clinical pharmacists perceived few barriers in their cognition of pharmaceutical care including the importance (2.8%) and the basic content (7.8%) of pharmaceutical care. And 47.4% and 44.7% of clinical pharmacists who attributed serious barriers to pharmaceutical care to a lack of financial compensation (e.g., pharmaceutical care fees) and insufficient various pharmaceutical workers to support pharmaceutical care, respectively. Moreover, the barriers in time constraints were serious, which included the lack of dedicated time for conducting pharmaceutical care (38.4%) and further education (33.5%).

**TABLE 2:** Differences in the frequency of various pharmaceutical care barriers.

Pharmaceutical care barriers	Agreement
Don’t understand the content	7.8%
Pharmaceutical care is not important	2.8%
Not confident	8.8%
No financial compensation	47.4%
Insufficient pharmaceutical knowledge	16.8%
Insufficient clinical medical knowledge	27.6%
Insufficient electronic information skills	24.6%
Not actively introducing to patients	18.6%
Electronic management system barriers	24.8%
Insufficient pharmaceutical workers	44.7%
Lack of regulations in medical institution	15.2%
Lack of dedicated place	31.5%
Lack of dedicated time	38.4%
Lack of electronic information system	31.6%
Non-standardized services and documents	28.0%
Self-identified as a non-health care provider	14.2%
Lack of communication with doctors	20.4%
Lack of communication with other healthcare providers	21.5%
Unable to get medical information	9.8%
Unable to modify the patient’s treatment plan	38.1%
Lack of opportunities for further education	23.2%
Lack of time for further education	33.5%
Lack of leadership support from medical institutions	20.0%
Lack of department leadership support	12.7%
Lack of legal and institutional support	29.0%

The regression results were found in Table 3 and Supplementary Tables S1–S4. None of independent variables were removed for suspected multicollinearity (Supplementary Table S5). Those models were relatively robust, and model 3 had a better explanation of the dependent variable than the others.

**TABLE 3:** Results of multiple linear regression (Model 3)^a^

	Role conflict			Role ambiguity		
Prob > F	0.0000			0.0000		
R-squared	0.2032			0.105		
Adj R-squared	0.1785			0.0773		
Root MSE	6.53			4.7706		

item	Role conflict			Role ambiguity		
	Coef	P	95% CI	Coef	P	95% CI
Age	0.037	0.420	[−0.053,0.127]	0.024	0.478	[−0.042,0.090]
Working years	0.040	0.323	[−0.039,0.118]	0.038	0.198	[−0.020,0.095]
Children	−0.048	0.946	[−1.439,1.343]	0.320	0.537	[−0.696,1.336]
Gender	0.585	0.136	[−0.184,1.353]	−0.218	0.445	[−0.780,0.343]
Education (ref = Below undergraduate)						
Undergraduate	1.689b	0.019	[0.278,3.100]	−0.780	0.138	[−1.811,0.251]
Master’s degree	2.781c	0.000	[1.233, 4.329]	−0.693	0.229	[−1.824,0.438]
PhD	2.679	0.208	[−1.490,6.848]	−1.029	0.507	[−4.075,2.017]
Marital status (ref = Unmarried)						
Married	−1.164	0.144	[−2.727,0.399]	0.168	0.774	[−0.974,1.309]
Other	−2.027	0.412	[−6.872, 2.818]	0.327	0.856	[−3.212,3.867]
Technical title (ref = junior title)						
Intermediate title	0.367	0.431	[−0.546,1.280]	0.305	0.370	[−0.362,0.972]
Deputy senior title	0.637	0.417	[−0.904, 2.178]	−0.048	0.933	[−1.174,1.078]
Positive senior title	−2.266	0.132	[−5.219, 0.687]	−2.331b	0.034	[−4.489,−0.174]
Don’t understand the content	1.237	0.089	[−0.190, 2.663]	−0.346	0.515	[−1.388,0.696]
Pharmaceutical care is not important	3.459d	0.004	[1.098, 5.819]	−1.322	0.133	[−3.046,0.403]
Not confident	1.451b	0.045	[0.035,2.868]	−0.031	0.952	[−1.066,1.004]
No financial compensation	0.985b	0.016	[0.186,1.785]	0.595b	0.046	[0.011,1.179]
Insufficient communication skills	1.761d	0.004	[0.572,2.949]	0.013	0.977	[−0.855,0.881]
Insufficient pharmaceutical knowledge	1.148	0.084	[−0.155,2.450]	−0.884	0.069	[−1.835,0.068]
Insufficient clinical medical knowledge	0.237	0.670	[−0.855, 1.329	−0.414	0.309	[−1.212,0.384]
Insufficient electronic information skills	0.579	0.282	[−0.476,1.634]	0.365	0.353	[−0.406,1.135]
Not actively introducing to patients	−0.005	0.992	[−1.020,1.009]	0.343	0.365	[−0.399,1.084]
Electronic management system barriers	0.795	0.140	[−0.261,1.850]	−0.580	0.140	[−1.351,0.191]
Insufficient pharmaceutical workers	1.174b	0.010	[0.282,2.066]	0.096	0.774	[−0.556,0.747]
Lack of regulations in medical institution	−0.093	0.880	[−1.296, 1.111]	−0.751	0.094	[−1.630,0.128]
Lack of dedicated place	−0.335	0.516	[−1.346,0.677]	−0.223	0.554	[−0.962,0.516]
Lack dedicated time	−0.216	0.665	[−1.193,0.761]	−0.852b	0.019	[−1.565,−0.138]
Lack of electronic information system	−0.370	0.465	[−1.364,0.624]	0.523	0.158	[−0.203, 1.249]
Non-standardized services and documents	2.120c	0.000	[1.099,3.141]	−1.117d	0.003	[-1.863,-0.371]
Self-identified as a non-health care provider	−0.086	0.881	[−1.214,1.042]	−0.209	0.619	[−1.033,0.615]
Lack of communication with doctors	0.785	0.260	[−0.583,2.153]	0.707	0.165	[−0.292,1.707]
Lack of communication with other healthcare providers	0.388	0.574	[−0.963,1.739]	−0.875	0.082	[−1.862,0.112]
Lack of communication with patients	0.036	0.950	[−1.094,1.166]	0.457	0.278	[−0.369,1.282]
Unable to get medical information	−0.203	0.765	[−1.536,1.131]	0.243	0.625	[−0.731,1.217]
Unable to modify the patient’s treatment plan	−0.365	0.395	[−1.208,0.477]	0.255	0.416	[−0.360,0.871]
Lack of opportunities for further education	0.193	0.723	[−0.878,1.264]	0.019	0.961	[−0.763,0.802]
Lack of time for further education	0.439	0.361	[−0.503,1.381]	−0.537	0.126	[−1.225,0.151]
Lack of leadership support from medical institutions	1.151	0.076	[−0.119,2.421]	−1.055b	0.026	[−1.983,−0.127]
Lack of department leadership support	−0.166	0.826	[−1.645,1.314]	−0.406	0.461	[−1.487,0.674]
Lack of legal and institutional support	0.683	0.160	[−0.271,1.637]	−0.156	0.661	[−0.853,0.541]

^a^ Coef = coefficient, CI, confidence interval; MSE, Mean mean-square error.

^b^ *p* < 0.05.

^c^ *p* < 0.001.

^d^ *p* < 0.01.

The pharmaceutical care barriers that had significant impact on clinical pharmacists’ role conflict included: clinical pharmacists’ (1) unawareness of the importance of pharmaceutical care, (2) lacking confidence in pharmaceutical care, and 3) insufficient communication skills; 4) the lack of financial compensation for pharmaceutical care; 5) insufficient various pharmaceutical workers; and 6) the failure to standardize the service procedures and contents of related documents in healthcare institutions. The coefficients were 3.459(*p* < 0.01), 1.451(*p* < 0.05), 1.761(*p* < 0.01), 0.985(*p* < 0.05), 1.174(*p* < 0.05), and 2.120 (*p* < 0.001), respectively.

In pharmaceutical care barriers, the lack of 1) financial compensation and 2) dedicated time for pharmaceutical care; 3) the failure to standardize the service procedures and contents of related documents in healthcare institutions; 4) the lack of support from healthcare institution leaders had significant impact on role ambiguity of clinical pharmacists, and the coefficients were 0.595(*p* < 0.05), −0.852(*p* < 0.05), −1.117(*p* < 0.01), −1.055(*p* < 0.05), respectively.

As for the control variables, both the degree of role conflict and role ambiguity increased as the educational level of clinical pharmacist increased. The degree of role conflict and role ambiguity did not always change stably or in the same direction. For example, with the promotion of the titles, the degree of role conflict increased, however, when a senior title was reached, the degree of role conflict decreased. Moreover, with the working years increasing, the degree of role conflict increased, while the role ambiguity decreased.

## 4 Discussion

This study analyzed the *status quo* of pharmaceutical care barriers among clinical pharmacists in China through field research. On this basis, a regression model was used to analyze the impact of pharmaceutical care barriers on role ambiguity and role conflict. According to the survey results, there were various barriers among Chinese clinical pharmacists in terms of providing pharmaceutical care and some of those barriers affected role ambiguity and role conflict significantly.

### 4.1 Common pharmaceutical care barriers

The lack of financial compensation is the most common pharmaceutical care barrier perceived by clinical pharmacists. A survey in United States in 2007 found that compensation was a barrier to outpatient pharmacists implementing medication therapy management (Lounsbery et al., 2009). Another study conducted in 2014 also showed that salary was one of the reasons affecting the implementation of pharmaceutical care in Chinese hospitals (Penm et al., 2014b). Despite the fact that the Chinese health authorities has issued a document supporting the introduction of additional fees for pharmaceutical care since 2010 (Minister of Health of the People’s Republic of China, 2010) and this policy has been carried out as pilot in a few areas (Guo et al., 2020). A remuneration system that reflects the value of pharmacists’ work in China has not been established. At present, clinical pharmacists are providing pharmaceutical care without financial support in China and only 18.1% of hospitals charge for pharmaceutical care in practice (Guo et al., 2020). In 2013, the International Pharmaceutical Federation argued that unpaid pharmaceutical care is unsustainable. It has been reported in previous studies that salary of pharmacists is one of the most integral motivator factors that could significantly impact the performance of pharmacists (Slimane, 2017; Carvajal and Popovici, 2018). Mature reimbursement mechanisms for pharmaceutical care have been established overseas, such as the Medscheck program (Ontario Ministry of Health and Long-Term Care, 2008) implemented in Ontario, Canada in 2007 and the Medicare Part D Medication Therapy Management Program (Touchette et al., 2006) implemented in the United States in 2006. Therefore, Chinese health authorities and hospitals should explore reimbursement mechanisms for the pharmaceutical care. For example, measures such as subsidizing clinical pharmacists who provide pharmaceutical care and charging for pharmaceutical care (Suxin, et al., 2020; Wan et al., 2022) may provide an incentive for clinical pharmacists to provide high quality pharmaceutical care.

Insufficient various pharmaceutical workers to support pharmaceutical care is also a commonly perceived barrier by clinical pharmacists. The number of pharmacists employed in the hospital setting has been reported as a main contributing factor to hospital pharmaceutical care development in Asian (Penm et al., 2014b; Willems et al., 2005) and European (Willems et al., 2005) countries. According to the Regulations on the Administration of Pharmaceutical Care in Hospitals published in 2011, public hospitals should have at least 8% of the total number of health professionals specialized in pharmaceutical care. The average proportion of the number of pharmacists to the total number of health professionals in 2019 is below the target set by the government in 2011 (Li et al., 2020a). The understaffing of pharmacists may also be related to the “zero mark-up policy” for drugs in the public hospitals in China. The pharmacy department becomes a non-profit sector when the mark-up on drugs is removed and hospitals may thereby reduce the number of pharmacists. The role of pharmacists in providing patient-centered pharmaceutical care in China is still at a developmental stage. Therefore, the recognition of pharmaceutical care in public hospitals needs to be strengthened in order to develop pharmaceutical care in China. In addition, the proportion of students majored in clinical pharmacy in China is relatively low in the overall pharmacy students, making it difficult to provide hospitals with adequate candidate pharmacists (Jie et al., 2020). Despite the shortage of pharmacists within hospitals in China, clinical pharmacy graduates are not qualified to enter high level hospitals limited by many factors such as academic level, competence structure and health management policies (Wenbing, 2022). The education and training of highly qualified personnel in clinical pharmacy needs to be strengthened as a long-term effort.

Unable to modify the patient’s treatment plan is also a significant pharmaceutical care barrier perceived by clinical pharmacists. Policy support is a strong incentive for the implementation of pharmaceutical care (Penm et al., 2014b). However, there is currently no policy supporting the role of pharmacists in pharmaceutical care in China. The pharmacists are marginalized in the multidisciplinary team in China (Yujing et al., 2017). Unlike countries such as the United States and Canada, Chinese clinical pharmacists do not have prescriptive authority (Liu and Chen, 2010; Onatade et al., 2017), making it challenging for pharmacists to be involved in therapeutic interventions. Therefore, the rights, duties and status of clinical pharmacists need to be specified at the legal level in order to help clinical pharmacists to promote rational drug use.

### 4.2 Pharmaceutical care barriers that had significant impact on both role conflict and role ambiguity

The barrier of lacking of financial compensation for pharmaceutical care increased the role conflict but reduced the role ambiguity. There have not yet issued any guidance on the fee schedule for pharmaceutical care in China. Clinical pharmacists are expected to take on multiple responsibilities and roles. However, if the corresponding financial compensation for pharmaceutical care is missing, the motivation of clinical pharmacists to carry out pharmaceutical care will be reduced. On the one hand, the reduction in motivation may cause clinical pharmacists to neglect the pharmaceutical care, therefore reducing role ambiguity in their work. On the other hand, it causes incompatible role expectations between clinical pharmacists themselves and those stakeholders who expected to access (for example, patients) or develop (such as policymakers) pharmaceutical care, leading to role conflict. Therefore, in order to overcome barriers to pharmaceutical care and reduce role conflict of clinical pharmacists, we should explore a compensation mechanism for pharmaceutical care based on the experience of the pilot pharmaceutical care fee and the successful practices of other countries.

Non standardization of the service procedures and contents of related documents of medical institutions increased the degree of both role conflict and ambiguity of clinical pharmacists. Nicholson and Goh have suggested that formal process management is negatively associated with role ambiguity and role conflict (Nicholson and Goh, 1983; Rai, 2016). The lack of standard service procedures and documents was not conducive to the consensus of expectations of all parties, which may lead to role conflict. Moreover, the incompatible and inconsistent expectations to clinical pharmacists may make them unable to deal with these expectations, which make them unclear about their work requirements and result in role ambiguity. The standardized medical documents are important resource for clinical pharmacists (Penm et al., 2014b), which are conductive to ensure that all patients receive appropriate patient-centered pharmaceutical care. We should establish the pharmaceutical care implementation guidelines and other management rules, such as standardized pharmaceutical care implementation protocols within the hospitals, which will help define the role of the clinical pharmacists and provide support on how to implement pharmaceutical care.

### 4.3 Pharmaceutical care barriers that had significant influence on either role conflict or role ambiguity

The lack of leaders’ support for pharmaceutical care could increase role ambiguity, which was consistent with the conclusion of previous studies that leaders’ structuring behaviors in health services can alleviate role ambiguity (House and Rizzo, 1972; Stout and Posner, 1984). Moreover, leadership support (e.g., emphasis on personal development, fault tolerance, supervisory support) in an organizational setting will reduce role ambiguity (House and Rizzo, 1972). The leader of pharmacy department could give the personal experience (Wenqian et al., 2013) to clinical pharmacists, helping them correctly understand the role of the clinical pharmacists as soon as possible and alleviate the role ambiguity. Previous studies have reported that the support of the director of pharmacy for the work of clinical pharmacists will influence the implementation of clinical pharmaceutical care (Penm et al., 2014b; Wenqian et al., 2013). However, in China, pharmaceutical care in most hospitals have not yet as thoroughly recognized as they are in developed countries such as the US, the UK and Australia. In this context, the role and status of pharmacists in the rational drug use has long been overlooked. China has already promoted the rational drug use and strengthened the status of pharmacists by setting up Pharmacy and Therapeutics Committee (Zheng et al., 2013) and piloting Chief Pharmacist System (Yang et al., 2022). Enhancing awareness of pharmaceutical care among others, especially hospital leaders, and supporting clinical pharmacists with resources such as opportunities of training and continuing education, dedicated time and space for pharmaceutical care, will help clinical pharmacists reduce role ambiguity and promote them to provide higher-quality pharmaceutical care.

The lack of dedicated time to carry out pharmaceutical care could also increase role ambiguity. According to organizational role theory, the organization will assign job roles to each employee (Wickham and Parker, 2007). The lack of dedicated time, the third major barrier perceived by Chinese clinical pharmacists, threatens the normalization of job role assignments and employee’s role perception, therefore leading to role ambiguity. A study in Pakistan similarly reported that lack of time was one of the barriers to implementing pharmaceutical care (Murtaza et al., 2015). Compared to the United States, where pharmacy staff work at least 8 hours a day in pharmaceutical care (Pedersen et al., 2019), however, only 40% of clinical pharmacists in tertiary hospitals in China spend 80% of their working time on clinical pharmacy-related work (Guo et al., 2020). Besides, there are still some clinical pharmacists who are mainly engaged in non-clinical pharmacy work such as drug dispensing and drug supply (Li et al., 2018). The utilization of the pharmacy technicians and machines may free clinical pharmacists from non-clinical pharmacy work.

This study also indicated that inadequate staffing of various pharmaceutical workers in medical institutions could increase the role conflict of clinical pharmacists. Heavy workload and insufficient pharmaceutical staffs make clinical pharmacists assume multiple roles, including clinical and non-clinical roles. Moreover, incompatible expectations between roles may lead to role conflict (EY and YY, 2018). And findings from a study of nurses suggested that the longer the working hours per week, the deeper the degree of role conflict (Sanli et al., 2021). The inadequate staffing of pharmaceutical workers in medical institutions may lead to extended working hours for clinical pharmacists, therefore exacerbating role conflict.

Insufficient communication skills of clinical pharmacists could increase role conflict. The clinical pharmacists work in an organization and need to perform multiple roles in response to the different requirements from the outside (for example, the leaders, colleagues, and patients). Insufficient communication skills may have an impact on the coordination of clinical pharmacists with other teams or individuals and therefore lead to role conflict (Randolph and Posner, 1981). PC is a patient-centered practice and communication between pharmacists and patients is essential to encourage rational drug use and optimize treatment outcomes (Aljumah and Hassali, 2015). Similarly, other healthcare providers also play an important role in the provision of PC and pharmacists should build strong partnerships with them in order to design appropriate care plans for patients’ medication-related problems (El Hajj et al., 2016). However, communication and other clinical skills are less cultivated in clinical pharmacy education curriculum in China. Chinese undergraduate education in clinical pharmacy takes 5 years and consists of pharmacy and basic medicine curriculum and clinical practice. However, the curriculum varies from one university to another and no uniform standards or guidelines have been set. The pharmacy and basic medicine curriculum in China are detached from the clinic and based on the chemistry curriculum and experiment. Moreover, courses related to doctor-patient communication, clinical medicine and medical ethics are missing in their curriculum in some universities. Take the curriculum of the 5-year clinical pharmacy at Shenyang Pharmaceutical University as an example, the courses of clinical practice are almost blank and the only course related to clinical communication is not a compulsory course (Yujing et al., 2017). In clinical practice training, clinical pharmacists are trained as generalists with both non-clinical duties and clinical duties. Therefore, the communication skills and clinical practice skills of pharmacists are inadequate and clinical pharmacists are not competent enough to take on the professional responsibility of pharmaceutical care. The curriculum of the clinical pharmacy profession in China needs further improvement. At present, strengthening students’ knowledge related to pharmacotherapy and cultivating clinical practice skills have become an important topic in the training of clinical pharmacy professionals in China. And to improve the *status quo*, it is significant to distinguish the clinical pharmacists from other pharmaceutical professionals by setting up core courses in medicine theory and clinical practice, basic courses in pharmacy and other courses in general studies (Fan et al., 2023), eliminate role ambiguity and role conflict for clinical pharmacists by setting up healthcare team awareness sessions and integrate hospital pharmacy practice throughout the teaching process to equip clinical pharmacy graduates with good communication and clinical practice skills.

### 4.4 Covariate variables

In this study, education background had a significantly positive impact on the clinical pharmacists’ role conflict, which was different from a previous study suggesting that education was helpful to alleviate role conflict of nurses (Wells, 2021). We usually think that with the improvement of educational level, the healthcare workers would be more competent to take on responsibilities and cope with uncertainty at work. However, clinical pharmacists, a group that has developed recently in China, have fewer clear responsibilities compared to nurses. The higher the education level of clinical pharmacists, the more complex the expectations of the healthcare institutions. When they cannot meet multiple incompatible job requirements, the role conflict may become serious. In view of this, clinical pharmacists need to constantly update their clinical knowledge and skills to improve their ability and keep pace with the evolving pharmaceutical care. And healthcare institutions can reduce clinical pharmacists’ role conflict by staffing enough highly educated clinical pharmacists and developing standardized working documents to assign them a reasonable workload.

Previous study had suggested that working years had a negative impact on role ambiguity and role conflict as a result of improvement in job perception and work competency (Ping et al., 2009). However, Celia (Wells, 2021) suggested that there was no significant relationship between more working years and role ambiguity and role conflict. This study showed a similar result that the effect of working years on role conflict and ambiguity of clinical pharmacists was not significant. The work mode and content of clinical pharmacists are in the stage of reform and exploration in China. The interference of changing job instructions and requirements may hinder the improvement of work perception and mitigate the impact of the working years on role ambiguity and role conflict. Therefore, the authorities need to develop relatively clear and somewhat stable responsibilities for clinical pharmacists.

### 4.5 Limitations

This study has some limitations. Firstly, the Chinese version of the Role Ambiguity and Role Conflict Scale specific to clinical pharmacists was not retrieved for this study. However, we found that the scale had acceptable reliability and validity among Chinese clinical pharmacists through pre-test. Secondly, because this was not a longitudinal study, we did not repeatedly measure the relationship between clinical pharmacists’ role conflict, role ambiguity and pharmaceutical care barriers. Therefore, biases caused by time factors might be ignored. Thirdly, since all of the participants are Chinese, the applicability of the findings to other countries remains to be verified. Fourthly, the measurement tool of pharmaceutical care barriers used in our study was not widely acknowledged in China. However, multiple rounds of expert consultation was conducted to develop the questionnaire and the results showed that it is able to meet the research needs of this study by capturing as comprehensively as possible the pharmaceutical care barriers that clinical pharmacists in China may confront in hospitals.

## 5 Conclusion

This study indicated that some of the pharmaceutical care barriers might have a negative effect on clinical pharmacists’ role conflict and role ambiguity. Based on the results of the above research, we recommended that several tasks need to be implemented by different sectors to promote the implementation of pharmaceutical care, including appropriate clinical pharmacy education, cost reimbursement mechanisms, hospital recognition, clear responsibilities, standardized service documents and supportive health policies and resources. Future research should explore the specific mechanism of how various pharmaceutical care barriers affect clinical pharmacists’ role conflict and role ambiguity, focus on how to reduce role conflict and role ambiguity of clinical pharmacists and enhance the quality of pharmaceutical care.

## Data Availability

The original contributions presented in the study are included in the article/Supplementary Material, further inquiries can be directed to the corresponding author.

## References

[B1] Aljumah K. Hassali M. A. (2015). Impact of pharmacist intervention on adherence and measurable patient outcomes among depressed patients: A randomised controlled study. Bmc Psychiatry 15, 219. 10.1186/s12888-015-0605-8 26376830 PMC4574071

[B2] Allemann S. S. Van Mil J. W. F. Botermann L. Berger K. Griese N. Hersberger K. E. (2014). Pharmaceutical care: The PCNE definition 2013. Int. J. Clin. Pharm. 36, 544–555. 10.1007/s11096-014-9933-x 24748506

[B3] Allison R. Simone P. (2013). How physician and community pharmacist perceptions of the community pharmacist role in Australian primary care influence the quality of collaborative chronic disease management. Qual. Prim. care 21, 105–111. 23735691

[B4] Avery A. J. Rodgers S. Cantrill J. A. Armstrong S. Cresswell K. Eden M. (2012). A pharmacist-led information technology intervention for medication errors (PINCER): A multicentre, cluster randomised, controlled trial and cost-effectiveness analysis. Lancet 379, 1310–1319. 10.1016/S0140-6736(11)61817-5 22357106 PMC3328846

[B5] Carvajal M. J. Popovici I. (2018). Gender, age, and pharmacists' job satisfaction. Pharm. Practice-Granada 16, 1396. 10.18549/PharmPract.2018.04.1396 PMC6322984 30637036

[B6] Dawoud D. Griffiths P. Maben J. Goodyer L. Greene R. (2011). Pharmacist supplementary prescribing: A step toward more independence? Res. Soc. Adm. Pharm. 7, 246–256. 10.1016/j.sapharm.2010.05.002 21272547

[B7] Ding H. Song Y. Wu N. Zheng X. Wei Q. Sun Y. (2022). Impact of individualized pharmaceutical care on efficacy and safety of opioid-tolerant outpatients with cancer pain: A multicenter randomized controlled trial. Ann. Transl. Med. 10, 989. 10.21037/atm-22-4091 36267757 PMC9577747

[B8] Doloresco F. Vermeulen L. C. (2009). Global survey of hospital pharmacy practice. Am. J. Health-System Pharm. 66, S13–S19. 10.2146/ajhp080674 19233967

[B9] Dong-Ning Y. Yuan-Kai H. Xiao-Yu X. (2019). National survey on clinical pharmacy services of second-level hospitals in China: Part 2. Analysis on the status quo of clinical pharmaceutical services. Chin. Pharm. J. 54, 150–157.

[B10] Ds L. Yh W. Ss W. (2017). Thinking and suggestion on positioning of clinical TCM pharmacists and the cultivation of post service ability. J. China Pharm. 28, 5170–5173.

[B11] El Hajj M. S. Al-S. A. E. E. D. Khaja M. (2016). Qatar pharmacists' understanding, attitudes, practice and perceived barriers related to providing pharmaceutical care. Int. J. Clin. Pharm. 38, 330–343. 10.1007/s11096-016-0246-0 26758716

[B12] Ey C. Yy H. (2018). Survey on the cognition of medical staff to clinical pharmacy work in primary hospitals. China Pharm. 21, 1041–1043.

[B13] Fan C. Pang R. Yao W. (2023). Exploration of reform paths of high-level talents training in clinical pharmacy. China Pharm. 34, 746–751.

[B14] Farris K. B. Fernandez-Llimos F. Benrimoj S. I. (2005). Pharmaceutical care in community pharmacies: Practice and research from around the world. Ann. Pharmacother. 39, 1539–1541. 10.1345/aph.1G049 16014373

[B15] Foppa A. A. Chemello C. Vargas-Pelaez C. M. Farias M. R. (2016). Medication therapy management service for patients with Parkinson's disease: A before-and-after study. Neurology Ther. 5, 85–99. 10.1007/s40120-016-0046-4 PMC4919135 27271736

[B16] Gallagher J. Mccarthy S. Byrne S. (2014). Economic evaluations of clinical pharmacist interventions on hospital inpatients: A systematic review of recent literature. Int. J. Clin. Pharm. 36, 1101–1114. 10.1007/s11096-014-0008-9 25218003

[B17] Guirguis L. M. Chewning B. A. (2005a). Role theory: Literature review and implications for patient-pharmacist interactions. Res. Soc. Adm. Pharm. 1, 483–507. 10.1016/j.sapharm.2005.09.006 17138492

[B18] Guirguis L. M. Chewning B. A. (2005b). Role theory: Literature review and implications for patient-pharmacist interactions. Res. Soc. Adm. Pharm. RSAP 1, 483–507. 10.1016/j.sapharm.2005.09.006 17138492

[B19] Guo X. Yao D. Liu J. Huang Y. Wang Y. Yao W. (2020). The current status of pharmaceutical care provision in tertiary hospitals: Results of a cross-sectional survey in China. Bmc Health Serv. Res. 20, 518. 10.1186/s12913-020-05371-7 32513167 PMC7282101

[B20] Hadi M. A. Alldred D. P. Briggs M. Munyombwe T. Closs S. J. (2014). Effectiveness of pharmacist-led medication review in chronic pain management systematic review and meta-analysis. Clin. J. Pain 30, 1006–1014. 10.1097/AJP.0000000000000063 24480911

[B21] House R. J. Rizzo J. R. (1972). Role conflict and ambiguity as critical variables in a model of organizational behavior. Organ. Behav. Hum. Perform. 7, 467–505. 10.1016/0030-5073(72)90030-x

[B22] Hua M. Yali Z. Xiufang Q. Qing S. (2015). The reliability and validity of Chinese version of nurse's role conflict and ambiguity questionnaire. J. Nurs. Adm. 15, 3–5.

[B23] Jie L. Donging Y. Rong H. (2020). The necessity and possibility of transforming nonclinical pharmacists to clinical pharmacists. Chin. I Hosp. Pharm. 40, 1192–1195.

[B24] Jorgenson D. Laubscher T. Lyons B. Palmer R. (2014). Integrating pharmacists into primary care teams: Barriers and facilitators. Int. J. Pharm. Pract. 22, 292–299. 10.1111/ijpp.12080 24279947

[B25] Kahn R. L. Rosenthal R. A. (1964). Organizational stress: Studies in role conflict and ambiguity.

[B26] Kahn R. L. Rosenthal R. A. (1965). Organizational stress: Studies in role conflict and ambiguity.

[B27] Katoue M. G. Ker J. (2018). Implementing the medicines reconciliation tool in practice: Challenges and opportunities for pharmacists in Kuwait. Health Policy 122, 404–411. 10.1016/j.healthpol.2017.12.011 29475740

[B28] Li C. Xiaoyu X. Dong-Ning Y. (2018). National survey on clinical pharmacy services of tertiary hospitals in China: Part 3. Status quo of clinical pharmacy service provision. Chin. Pharm. J. 53, 837–842.

[B29] Li M. Cao M. Sun J. Jiang Y. Liu Y. (2020a). Pharmaceutical care in Chinese public tertiary hospitals: Findings from the 4th national healthcare improvement initiative survey. Hum. Resour. Health 18, 31. 10.1186/s12960-020-00473-z 32345325 PMC7189700

[B30] Li W. Lin G. Xu A. Huang Y. XI X. (2020b). Role ambiguity and role conflict and their influence on responsibility of clinical pharmacists in China. Int. J. Clin. Pharm. 42, 879–886. 10.1007/s11096-020-01053-w 32405715

[B31] Liang-Jiang C. Yuan-Kai H. Xiao-Yu X. (2022). Literature analysis of the instruments measuring the barriers of pharmaceutical care. Chin. J. Hosp. Pharm. 42, 564–569.

[B32] Liu G. Chen R. (2010). Status and role of clinical pharmacists under new medical reform. China Pharm. 21, 2785–2791.

[B33] Lounsbery J. L. Green C. G. Bennett M. S. Pedersen C. A. (2009). Evaluation of pharmacists' barriers to the implementation of medication therapy management services. J. Am. Pharm. Assoc. 49, 51–58. 10.1331/JAPhA.2009.017158 19196597

[B34] Martin-Calero M. J. Machuca M. Murillo M. D. Cansino J. Gastelurrutia M. A. Faus M. J. (2004). Structural process and implementation programs of pharmaceutical care in different countries. Curr. Pharm. Des. 10, 3969–3985. 10.2174/1381612043382549 15579083

[B35] Ming-Xia W. Yue-Ping L. Shi-Yue Y. Wen L. (2014). The correlation between job burnout and medical safety of clinicians and its influencing factors. J. Fujian Med. Univ. Soc. Sci. Ed. 15, 22–25.

[B36] Minister Of Health Of The People'S Republic Of China (2010). Circular on the issuance of guidance on public hospital reform pilot.

[B37] Minister Of Health Of The People'S Republic Of China (2011). Regulations on pharmacy administration of medical institutions.

[B38] Murtaza G. Kousar R. Azhar S. Khan S. A. Mahmood Q. (2015). What do the hospital pharmacists think about the quality of pharmaceutical care services in a Pakistani province? A mixed methodology study. Biomed. Res. Int. 2015, 756180. 10.1155/2015/756180 25649021 PMC4306404

[B39] National Health Commission (2018a). Opinions on accelerating the high-quality development of pharmaceutical care.

[B40] National Health Commission (2018b). Statistical bulletin on the development of health care in China in 2018. Accessed Available at: http://www.gov.cn/guoqing/2020-04/29/content_5507528.htm.

[B41] National Health Commission (2021). Notice on the issuance of 5 standards for pharmacist-managed clinic services and so on in medical institutions.

[B42] Nicholson P. J. Goh S. C. (1983). The relationship of organization structure and interpersonal attitudes to role-conflict and ambiguity in different work environments. Acad. Manag. J. 26, 148–155. 10.2307/256141

[B43] Onatade R. Sawieres S. Veck A. Smith L. Gore S. Al-Azeib S. (2017). The incidence and severity of errors in pharmacist-written discharge medication orders. Int. J. Clin. Pharm. 39, 722–728. 10.1007/s11096-017-0468-9 28573438 PMC5541123

[B44] Ontario Ministry Of Health And Long-Term Care (2008). MedsCheck program summaries. Accessed Available at: http://www.health.gov.on.ca/en/common/system/default.aspx .

[B45] Pedersen C. A. Schneider P. J. Ganio M. C. Scheckelhoff D. J. (2019). ASHP national survey of pharmacy practice in hospital settings: Monitoring and patient education-2018. Am. J. Health-System Pharm. 76, 1038–1058. 10.1093/ajhp/zxz099 31361881

[B46] Penm J. Li Y. Zhai S. Hu Y. Chaar B. Moles R. (2014a). The impact of clinical pharmacy services in China on the quality use of medicines: A systematic review in context of China's current healthcare reform. Health Policy Plan. 29, 849–872. 10.1093/heapol/czt067 24056897

[B47] Penm J. Moles R. Wang H. Li Y. Chaar B. (2014b). Factors affecting the implementation of clinical pharmacy services in China. Qual. HEALTH Res. 24, 345–356. 10.1177/1049732314523680 24562375

[B48] Perrot S. Cittee J. Louis P. Quentin B. Robert C. Milon J.-Y. (2019). Self-medication in pain management: The state of the art of pharmacists' role for optimal Over-The-Counter analgesic use. Eur. J. Pain 23, 1747–1762. 10.1002/ejp.1459 31349370

[B49] Ping Y. Li-Ying W. Hui-Cai W. Mei-Xin W. Shu-Juan N. (2009). Role conflict and role ambiguity among head nurses and the influencing factors. J. Nurs. Sci. 24, 11–13.

[B50] Prc N. H. C. O. T. (2018). Opinions on accelerating the quality development of pharmacy services.

[B51] Rai G. S. (2016). Minimizing role conflict and role ambiguity: A virtuous organization approach. Hum. Serv. Organ. Manag. Leadersh. Gov. 40, 508–523. 10.1080/23303131.2016.1181594

[B52] Randolph W. A. Posner B. Z. (1981). Explaining role-conflict and role ambiguity via individual and interpersonal variables in different job categories. Pers. Psychol. 34, 89–102. 10.1111/j.1744-6570.1981.tb02180.x

[B53] Raosoft (2008). An online sample size calculator. Accessed Available: http://www.raosoft.com/samplesize.html .

[B54] Rizzo H. R. Si L. Lirtzman S. I. (1970). Role conflict and ambiguity in complex organizations. Adm. Sci. Q. 15, 150–163. 10.2307/2391486

[B55] Rovithis M. Linardakis M. Rikos N. Merkouris A. Patiraki E. Philalithis A. (2009). Role conflict and role ambiguity among head nurses and the influencing factors. J. Nurs. Sci. 24, 11–13.

[B56] Sanli D. Cimen M. U. Isler N. Turgut N. (2021). Role conflict and role ambiguity in pediatric general duty and intensive care unit nurses. CYPRUS J. Med. Sci. 6, 162–170. 10.5152/cjms.2021.2153

[B57] Scahill S. Harrison J. Sheridan J. (2009). The ABC of New Zealand's ten year vision for pharmacists: Awareness, barriers and consultation. Int. J. Pharm. Pract. 17, 135–142. 10.1211/ijpp.17.03.0003 20218244

[B58] Seaton T. L. Bown-Luzier A. Cooke C. E. Deconinck J. F. Dupuis R. E. Force R. W. (2000). Pharmacotherapy: A position statement of the American college of clinical pharmacy. Pharmacotherapy 20, 487–490. 10.1592/phco.20.5.487.35054 10772381

[B59] Senatra P. T. (1980). ROLE-CONFLICT, role ambiguity, and organizational-climate in a public accounting firm. Account. Rev. 55, 594–603.

[B60] Shao H. Chen G. Zhu C. Chen Y. Liu Y. He Y. (2017). Effect of pharmaceutical care on clinical outcomes of outpatients with type 2 diabetes mellitus. Patient Prefer. Adherence 11, 897–903. 10.2147/PPA.S92533 28507433 PMC5428753

[B61] Slimane N. S. B. (2017). Motivation and job satisfaction of pharmacists in four hospitals in Saudi arabia. J. Health Manag. 19, 39–72. 10.1177/0972063416682559

[B62] Stout J. K. Posner J. L. (1984). STRESS, role ambiguity, and role-conflict. Psychol. Rep. 55, 747–753. 10.2466/pr0.1984.55.3.747 6522540

[B63] Suxin W. Wei F. Daoqiu H. (2020). Investigation and study on present situation of clinical pharmaceutical care in 39 medical institutions in chongqing. China Pharm. 31, 12–17.

[B64] Taylor S. Cairns A. Glass B. (2020). Role theory: A framework to explore health professional perceptions of expanding rural community pharmacists' role. PHARMACY 8, 161. 10.3390/pharmacy8030161 32887322 PMC7559310

[B65] Teh P. L. Yong C.-C. Lin B. (2014). Multidimensional and mediating relationships between tqm, role conflict and role ambiguity: A role theory perspective. TOTAL Qual. Manag. Bus. Excell. 25, 1365–1381. 10.1080/14783363.2012.733266

[B66] Touchette D. R. Burns A. L. Bough M. A. Blackburn J. C. (2006). Survey of medication therapy management programs under medicare part D. J. Am. Pharm. Assoc. 46, 683–691. 10.1331/1544-3191.46.6.683.touchette 17176683

[B67] Tunc T. Kutanis R. O. (2009). Role conflict, role ambiguity, and burnout in nurses and physicians at a University hospital in Turkey. Nurs. Health Sci. 11, 410–416. 10.1111/j.1442-2018.2009.00475.x 19909450

[B68] Vulaj V. Hough S. Bedard L. Farris K. Mackler E. (2018). Oncology pharmacist opportunities: Closing the gap in quality care. J. Oncol. Pract. 14, E403–E411. 10.1200/JOP.2017.026666 29298114

[B69] Wan C. Huang Y. Chen L. XI X. (2022). The influence of non-clinical pharmacists' understanding of and attitudes towards pharmaceutical care on their willingness to serve as clinical pharmacists in China. Bmc Health Serv. Res. 22, 484. 10.1186/s12913-022-07734-8 35413836 PMC9004027

[B70] Wells C. M. (2021). Factors influencing role ambiguity and role conflict among intensive care unit nurses providing end of life care. J. Nurs. Adm. 51, 620–625. 10.1097/NNA.0000000000001084 34789689

[B71] Wenbing Y. (2022). Accelerate the training of scientist pharmacists in a clinically oriented manner. Health News.

[B72] Wenqian B. (2013). Influence of social support on role conflict and role ambiguity of head nurses. Chin. Nurs. Res. 27, 1718–1719.

[B73] Wickham M. Parker M. (2007). Reconceptualising organisational role theory for contemporary organisational contexts. J. Manag. Psychol. 22, 440–464. 10.1108/02683940710757182

[B74] Willems L. Raymakers A. Sermeus W. Vleugels A. Laekeman G. (2005). Survey of hospital pharmacy practice in Flemish-speaking Belgium. Am. J. Health-System Pharm. 62, 321–324. 10.1093/ajhp/62.3.321 15719594

[B75] Xiao-Yu X. Dong-Ning Y. Yuan-Kai H. Xiao-Jing W. Yi-Tao W. Wen-Bing Y. (2018). National survey on clinical pharmacy services of tertiary hospitals in China: Part 4. Related personnel's attitudes toward clinical pharmacy services. Chin. Pharm. J. 53, 1123–1129.

[B76] Xie J. Li J. Wang S. Li L. Wang K. Duan Y. Liu Q. Zhong Z. (2014). The correlation between job burnout and medical safety of clinicians and its influencing factors. J. Fujian Med. Univ. Soc. Sci. Ed. 15, 22–25.

[B77] Xie C. Mu X. Hu Z. Wang W. Huang W. Huang G. (2020). Impact of pharmaceutical care in the orthopaedic department. J. Clin. Pharm. Ther. 45, 401–407. 10.1111/jcpt.13091 31800132

[B78] Yang R. Li Q. Hayat K. Zhai P. Liu W. Chen C. (2022). Views of pharmacists and government representatives toward the pilot Chief pharmacist system in Chinese hospitals: A multicenter exploratory qualitative study. Front. Public Health 10, 895649. 10.3389/fpubh.2022.895649 35784261 PMC9240424

[B79] Yu Y. Jingyi Z. Jing L. Ting X. (2016). Development of laws and regulations for clinical pharmacist in USA and Japan and its enlightenment for China. China Pharm. 19, 2128–2130.

[B80] Yuan-Kai H. Dong-Ning Y. Xiao-Yu X. Yi W. Xiao-Jing W. Yi-Tao W. (2019). National survey on clinical pharmacy services of second-level hospitals in China: Part 3. Analysis of attitudes of personnel involved in clinical pharmacy service. Chin. Pharm. J. 54, 245–252.

[B81] Yujing L. Yue Y. Jingyu Y. (2017). Analysis of the current situation of clinical pharmacy education in China. China University Teaching, 89–92.

[B82] Yy Z. Y, Y. L, M. Yq W. (2017). Investigation of the development of clinical pharmacy in 28 hospitals of Jiangsu province. J. China Pharm., 3341–3346.

[B83] Zheng Z. Yun Y. Yilan H. (2013). Investigation and analysis of the status quo of pharmaceutical administration in medical institutions of sichuan province. China Pharm. 24, 2980–2983.

