# Supplementary material for: Association of pharmaceutical care barriers and role ambiguity and role conflict of clinical pharmacists
Source: Front Pharmacol. 2023 May 9;14:1103255. doi: 10.3389/fphar.2023.1103255 (PMC10203618; doi:10.3389/fphar.2023.1103255)
Supplement: Supplementary file 1 [file Table1.DOCX]

Supplementary Material

| Clinical Pharmacist Questionnaire | | | |
| --- | --- | --- | --- |
| Dear clinical pharmacist:  This is a questionnaire about the pharmaceutical care barriers, role ambiguity and role conflict of clinical pharmacists in China. It aims to study the impact of pharmaceutical care barriers on the role ambiguity and role conflict of clinical pharmacists, so as to provide information for the development of clinical pharmacists in China. The survey results will be kept strictly confidential and used only for academic research and not for any commercial purpose.  The questions in the questionnaire are single-choice questions, multiple choice questions or blank questions. Please answer according to your actual situation and attitude. If you have any questions about the requirements of the questionnaire, please feel free to ask the investigator. Thank you for your support and cooperation. | | | |
| Full name of the hospital you work for: | | __________ | |
| Your contact information (phone number or email): | | __________ | |
| Your gender: | | □Male □Female | |
| Your age: | | __________ | |
| Your current marital status: | | □Unmarried □Married □Others (divorce, widowhood, etc.) | |
| The current number of your children: | | __________ | |
| Years of service in your current position or similar position | | __________ | |
| Your technical title | | □junior title □Intermediate title  □Deputy senior title □Positive senior title | |
| Your education | | □Below undergraduate □Undergraduate □Master's degree□PhD | |
| Your qualification as a clinical pharmacist is | | □Nationally-trained specialist clinical pharmacist  □Nationally-trained general clinical pharmacist  □Provincial-trained specialist clinical pharmacist  □Provincial-trained general pharmacist  □Untrained clinical pharmacist | |
| The name of the profession you are currently working in | | □Anti-Infective Program □Cardiovascular Medicine  □Respiratory Medicine □Gastroenterology  □Nephrology □Antitumor Medicine  □Organ Transplantation □ICU □Endocrinology  □Neurology □Other | |
| Your participation in training and qualification acquisition | | □Certificate of completion of clinical pharmacist training from the Ministry of Health  □Clinical pharmacist advanced training certificate  □Clinical pharmacist training teacher certificate of the Health Planning Commission  □Go abroad to participate in relevant clinical pharmacist training  □Other training | |
| In the rest of the questionnaire, please choose the option that best matches your perception, feeling or opinion at work. | | | |
| Regarding your current position rights and job content (Role Conflict and Role Ambiguity Scale): | | | |
| (1) I know what authority I have. | | □Strongly disagree □Largely disagree □Slightly disagree □Never mind □Slightly agree □Largely agree □Strongly agree | |
| (2) My work goals and objectives are clear and organized. | | □Strongly disagree □Largely disagree □Slightly disagree □Never mind □Slightly agree □Largely agree □Strongly agree | |
| (3) I think I have allocated my time appropriately. | | □Strongly disagree □Largely disagree □Slightly disagree □Never mind □Slightly agree □Largely agree □Strongly agree | |
| (4) I know what responsibilities I have to take. | | □Strongly disagree □Largely disagree □Slightly disagree □Never mind □Slightly agree □Largely agree □Strongly agree | |
| (5) I know exactly what others expect me to do. | | □Strongly disagree □Largely disagree □Slightly disagree □Never mind □Slightly agree □Largely agree □Strongly agree | |
| (6) What I need to do is explained very clearly. | | □Strongly disagree □Largely disagree □Slightly disagree □Never mind □Slightly agree □Largely agree □Strongly agree | |
| (7) What I need to do needs to be done in many different ways. | | □Strongly disagree □Largely disagree □Slightly disagree □Never mind □Slightly agree □Largely agree □Strongly agree | |
| (8) I need to accomplish something that is understaffed and difficult to accomplish. | | □Strongly disagree □Largely disagree □Slightly disagree □Never mind □Slightly agree □Largely agree □Strongly agree | |
| (9) I have to violate rules or policies to get some work done. | | □Strongly disagree □Largely disagree □Slightly disagree □Never mind □Slightly agree □Largely agree □Strongly agree | |
| (10) I work with two or more teams, and they operate in completely different ways. | | □Strongly disagree □Largely disagree □Slightly disagree □Never mind □Slightly agree □Largely agree □Strongly agree | |
| (11) I get two or more requests which may make me lose at least one of them. | | □Strongly disagree □Largely disagree □Slightly disagree □Never mind □Slightly agree □Largely agree □Strongly agree | |
| (12) What I do is easily accepted by one person but not by others. | | □Strongly disagree □Largely disagree □Slightly disagree □Never mind □Slightly agree □Largely agree □Strongly agree | |
| (13) I will be arranged to do things that do not have enough resources and materials to implement. | | □Strongly disagree □Largely disagree □Slightly disagree □Never mind □Slightly agree □Largely agree □Strongly agree | |
| (14) I will do things that I don't have to do. | | □Strongly disagree □Largely disagree □Slightly disagree □Never mind □Slightly agree □Largely agree □Strongly agree | |
| You have encountered the following barriers in your work: | | | |
| Pharmacist dimension | (1) You do not understand the basic content of pharmaceutical care (such as medication consultation, prescription review and comment, pharmaceutical monitoring, pharmaceutical rounds, etc.) | | □Disagree □Agree |
| Pharmacist dimension | (2) You think it is not important to provide pharmaceutical care | | □Disagree □Agree |
| Pharmacist dimension | (3) You don't have confidence in pharmaceutical care | | □Disagree □Agree |
| Financial dimension | (4) pharmaceutical care are not encouraged and supported through financial compensation (such as pharmacy service fees) | | □Disagree □Agree |
| Pharmacist dimension | (5) Due to insufficient communication skills, you are powerless in pharmaceutical care | | □Disagree □Agree |
| Pharmacist dimension | (6) Due to insufficient pharmaceutical knowledge, you are powerless in pharmaceutical care | | □Disagree □Agree |
| Pharmacist dimension | (7) Due to insufficient clinical medical knowledge, you are powerless in pharmaceutical care | | □Disagree □Agree |
| Pharmacist dimension | (8) Due to insufficient electronic information technology and data retrieval skills, you are powerless in pharmaceutical care | | □Disagree □Agree |
| Collaboration dimension | (9) You do not actively explain to patients what pharmaceutical care are, and at what levels pharmaceutical care can help patients | | □Disagree □Agree |
| Resource dimension | (10) The electronic management system of pharmaceutical care (such as hospital management system, pharmaceutical monitoring software) in your medical institution is difficult to use | | □Disagree □Agree |
| Resource dimension | (11) Your medical institution is understaffed with all kinds of pharmaceutical workers | | □Disagree □Agree |
| Social dimension | (12) Your medical institution lacks regulations for pharmaceutical care | | □Disagree □Agree |
| Resource dimension | (13) Your medical institution lacks a dedicated place to provide pharmaceutical care | | □Disagree □Agree |
| Resource dimension | (14) You lack dedicated time for pharmaceutical care | | □Disagree □Agree |
| Resource dimension | (15) Your medical institution lacks the electronic information system and prescription evaluation system needed to carry out pharmaceutical care | | □Disagree □Agree |
| Social dimension | (16) Service processes and related documentation in your medical institution have not been standardized | | □Disagree □Agree |
| Pharmacist dimension | (17) You do not consider yourself a "health care provider ", who provides health care directly to a patient | | □Disagree □Agree |
| Collaboration dimension | (18) You lack communication with doctors and their support | | □Disagree □Agree |
| Collaboration dimension | (19) You lack communication with other health care providers and their support | | □Disagree □Agree |
| Collaboration dimension | (20) You lack communication with the patient and their support | | □Disagree □Agree |
| Social dimension | (21) You cannot get patient medical information, including medical records, prescriptions, test results, etc. | | □Disagree □Agree |
| Social dimension | (22) You cannot modify the patient's treatment plan | | □Disagree □Agree |
| Resource dimension | (23) You lack opportunities for further education | | □Disagree □Agree |
| Resource dimension | (24) You lack time for further education | | □Disagree □Agree |
| Collaboration dimension | (25) Lack of support from the leaders of your medical institution for pharmaceutical care | | □Disagree □Agree |
| Collaboration dimension | (26) Lack of support from your department leader for pharmaceutical care | | □Disagree □Agree |
| Social dimension | (27) Lack of legal and institutional support for pharmaceutical care | | □Disagree □Agree |
