# Supplementary material for: Association of pharmaceutical care barriers and role ambiguity and role conflict of clinical pharmacists
Source: Front Pharmacol. 2023 May 9;14:1103255. doi: 10.3389/fphar.2023.1103255 (PMC10203618; doi:10.3389/fphar.2023.1103255)
Supplement: Supplementary file 2 [file Table2.docx]

Supplementary Material

# Supplementary Tables

**eTable 1**. F-test results of regression model (Model 1)

|  | Role conflict | Role ambiguity |
| --- | --- | --- |
| Prob > F | 0.0000 | 0.0000 |
| R-squared | 0.1861 | 0.0877 |
| Adj R-squared | 0.1688 | 0.0684 |
| Root MSE | 6.5684 | 4.7935 |

MSE = Mean square error

**eTable 2**. Results of multiple linear regression (Model 1) ^a^

| item | role conflict | | | role ambiguity | | |
| --- | --- | --- | --- | --- | --- | --- |
|  | coef | p | 95%CI | coef | p | 95%CI |
| Don’t understand the content | 1.313 | 0.071 | [-0.114,2.740] | -0.322 | 0.544 | [-1.364,0.719] |
| Pharmaceutical care is not important | 3.983^b^ | 0.001 | [1.643,6.323] | -1.533 | 0.078 | [-3.240,0.175] |
| Not confident | 1.179 | 0.102 | [-0.236,2.594] | -0.406 | 0.939 | [-1.073,0.992] |
| No financial compensation | 1.005^c^ | 0.014 | [0.206,1.805] | 0.581 | 0.051 | [-.002, 1.165] |
| Insufficient communication skills | 1.696^c^ | 0.005 | [0.506,2.887] | -0.080 | 0.856 | [-0.949,0.788] |
| Insufficient pharmaceutical knowledge | 1.067 | 0.108 | [-0.236,2.370] | -0.790 | 0.103 | [-1.741,0.161] |
| Insufficient clinical medical knowledge | 0.351 | 0.527 | [-0.738,1.439] | -0.387 | 0.339 | [-1.181,0.407] |
| Insufficient electronic information skills | 0.363 | 0.498 | [-0.688,1.414] | 0.358 | 0.360 | [-0.409,1.125] |
| Not actively introducing to patients | -0.042 | 0.936 | [-1.060,0.977] | 0.423 | 0.264 | [-0.320,1.167] |
| Electronic management system barriers | 0.762 | 0.156 | [-0.292,1.817] | -0.539 | 0.169 | [-1.309,0.230] |
| Insufficient pharmaceutical workers | 1.234^b^ | 0.007 | [0.342,2.125] | 0.032 | 0.924 | [-0.619,0.682] |
| Lack of regulations in medical institution | 0.097 | 0.875 | [-1.106,1.299] | -0.601 | 0.179 | [-1.478,0.277] |
| Lack of dedicated place | -0.345 | 0.505 | [-1.359,0.669] | -0.155 | 0.682 | [-0.894,0.585] |
| Lack dedicated time | -0.171 | 0.733 | [-1.150,0.809] | -0.876 | 0.016 | [-1.590,-0.161] |
| Lack of electronic information system | -0.383 | 0.450 | [-1.379,0.612] | 0.452 | 0.223 | [-0.275,1.179] |
| Non-standardized services and documents | 2.059^d^ | 0.000 | [1.037,3.080] | -1.091^b^ | 0.004 | [-1.837,-0.346] |
| Self-identified as a non-health care provider | -0.040 | 0.945 | [-1.173,1.093] | -0.232 | 0.582 | [-1.059,0.594] |
| Lack of communication with doctors | 0.667 | 0.341 | [-0.705,2.039] | 0.633 | 0.215 | [-0.369,1.634] |
| Lack of communication with other health care providers | 0.398 | 0.565 | [-0.958,1.755] | -0.885 | 0.080 | [-1.875,0.105] |
| Lack of communication with patients | -0.062 | 0.914 | [-1.193,1.068] | 0.375 | 0.373 | [-0.450,1.200] |
| Unable to get medical information | -0.222 | 0.744 | [-1.557,1.112] | 0.314 | 0.527 | [-0.660,1.288] |
| Unable to modify the patient's treatment plan | -0.488 | 0.257 | [-1.333,0.356] | 0.238 | 0.448 | [-0.378,0.855] |
| Lack of opportunities for further education | 0.159 | 0.771 | [-0.915,1.234] | 0.045 | 0.911 | [-0.739,0.829] |
| Lack of time for further education | 0.599 | 0.211 | [-0.341,1.538] | -0.476 | 0.174 | [-1.161,0.210] |
| Lack of leadership support from medical institutions | 1.332 | 0.040 | [0.060,2.604] | -1.060 | 0.025 | [-1.989, -0.132] |
| Lack of department leadership support | -0.167 | 0.825 | [-1.650,1.315] | -0.407 | 0.461 | [-1.489,0.675] |
| Lack of legal and institutional support | 0.628 | 0.197 | [-0.325,1.580] | -0.265 | 0.455 | [-0.960,0.431] |

Coef = coefficient, CI = confidence interval.

**eTable 3**. F-test results of regression model (Model 2)

|  | Role conflict | Role ambiguity |
| --- | --- | --- |
| Prob > F | 0.0000 | 0.0000 |
| R-squared | 0.1905 | 0.1008 |
| Adj R-squared | 0.1707 | 0.0788 |
| Root MSE | 6.561 | 4.7666 |

MSE = Mean mean-square error.

**eTable 4**. Results of multiple linear regression (Model 2) ^a^

| **item** | **role conflict** | | | **Role ambiguity** | | |
| --- | --- | --- | --- | --- | --- | --- |
|  | **coef** | **P** | **95%CI** | **coef** | **P** | **95%CI** |
| Don’t understand the content | 1.306 | 0.073 | [-0.123,2.735] | -0.317 | 0.549 | [-1.356,0.721] |
| Pharmaceutical care is not important | 4.038^b^ | 0.001 | [1.693,6.383] | -1.567 | 0.071 | [-3.270,0.137] |
| Not confident | 1.269 | 0.079 | [-0.146,2.684] | 0.008 | 0.987 | [-1.020,1.036] |
| No financial compensation | 0.984^c^ | 0.016 | [0.184,1.785] | 0.606^c^ | 0.041 | [0.025,1.188] |
| Insufficient communication skills | 1.759^b^ | 0.004 | [0.569,2.949] | 0.011 | 0.980 | [-0.854,0.876] |
| Insufficient pharmaceutical knowledge | 1.023 | 0.123 | [-0.279,2.326] | -0.827 | 0.087 | [-1.773,0.119] |
| Insufficient clinical medical knowledge | 0.327 | 0.556 | [-0.762,1.416] | -0.431 | 0.285 | [-1.222,0.360] |
| Insufficient electronic information skills | 0.388 | 0.471 | [-0.667,1.443] | 0.418 | 0.285 | [-0.348,1.185] |
| Not actively introducing to patients | -0.066 | 0.898 | [-1.084,0.952] | 0.357 | 0.345 | [-0.383,1.096] |
| Electronic management system barriers | 0.702 | 0.192 | [-0.354,1.758] | -0.564 | 0.149 | [-1.332,0.203] |
| Insufficient pharmaceutical workers | 1.236^b^ | 0.007 | [0.344,2.128] | 0.025 | 0.939 | [-0.622,0.673] |
| Lack of regulations in medical institution | -0.039 | 0.949 | [-1.245,1.166] | -0.739 | 0.098 | [-1.615,0.137] |
| Lack of dedicated place | -0.365 | 0.480 | [-1.379,0.648] | -0.208 | 0.579 | [-0.945,0.528] |
| Lack dedicated time | -0.138 | 0.781 | [-1.117,0.840] | -0.848^c^ | 0.019 | [-1.559,-0.138] |
| Lack of electronic information system | -0.350 | 0.490 | [-1.346,0.645] | 0.508 | 0.169 | [-0.216,1.231] |
| Non-standardized services and documents | 2.038^d^ | 0.000 | [1.015,3.061] | -1.104 | 0.004 | [-1.847,-0.360] |
| Self-identified as a non-health care provider | -0.016 | 0.978 | [-1.149,1.116] | -0.216 | 0.606 | [-1.039,0.606] |
| Lack of communication with doctors | 0.719 | 0.304 | [-0.653,2.091] | 0.694 | 0.172 | [-0.302,1.691] |
| Lack of communication with other health care providers | 0.418 | 0.546 | [-0.938,1.773] | -0.853 | 0.090 | [-1.837,0.132] |
| Lack of communication with patients | -0.015 | 0.979 | [-1.148,1.118] | 0.477 | 0.255 | [-0.345,1.300] |
| Unable to get medical information | -0.269 | 0.694 | [-1.607,1.069] | 0.239 | 0.629 | [-0.733,1.211] |
| Unable to modify the patient's treatment plan | -0.452 | 0.294 | [-1.297,0.393] | 0.282 | 0.368 | [-0.332,0.896] |
| Lack of opportunities for further education | 0.167 | 0.760 | [-0.907,1.241] | 0.037 | 0.925 | [-0.743,0.817] |
| Lack of time for further education | 0.535 | 0.265 | [-0.406,1.475] | -0.571 | 0.102 | [-1.254,0.113] |
| Lack of leadership support from medical institutions | 1.309 | 0.044 | [0.037, 2.582] | -1.082^c^ | 0.022 | [-2.006,-0.158] |
| Lack of department leadership support | -0.171 | 0.821 | [-1.654,1.312] | -0.375 | 0.494 | [-1.453,0.702] |
| Lack of legal and institutional support | 0.704 | 0.148 | [-0.250,1.657] | -0.195 | 0.581 | [-0.888,0.498] |
| Working years | 0.013 | 0.669 | [-0.046,0.072] | 0.064^b^ | 0.004 | [0.021,0.106] |
| Technical title (ref = junior title) |  |  |  |  |  |  |
| Intermediate title | 0.546 | 0.200 | [-0.290,1.382] | 0.457 | 0.140 | [-0.151,1.064] |
| Deputy senior title | 1.212 | 0.088 | [-0.179,2.604] | 0.108 | 0.834 | [-0.903,1.119] |
| Positive senior title | -1.415 | 0.332 | [-4.278,1.448] | -2.195^c^ | 0.039 | [-4.275,-0.115] |

^a^coef = coefficient, CI = confidence interval.

^b^p<0.01

^c^p<0.05

^d^p<0.001

**eTable 5**. Collinearity test ^e^

| **item** | **Tolerance** | **VIF** |
| --- | --- | --- |
| Age | 0.306 | 3.27 |
| Working years | 0.390 | 2.56 |
| Children | 0.358 | 2.79 |
| Sex | 0.941 | 1.06 |
| Education |  |  |
| Below undergraduate |  |  |
| Undergraduate | 0.271 | 3.67 |
| Master's degree | 0.259 | 3.86 |
| PhD | 0.866 | 1.16 |
| Marital status |  |  |
| Unmarried |  |  |
| Married | 0.369 | 2.71 |
| Other | 0.879 | 1.14 |
| Technical title |  |  |
| junior title |  |  |
| Intermediate title | 0.607 | 1.65 |
| Deputy senior title | 0.530 | 1.89 |
| Senior title | 0.768 | 1.30 |
| Don’t understand the content | 0.866 | 1.15 |
| Pharmaceutical care is not important | 0.841 | 1.19 |
| Not confident | 0.786 | 1.27 |
| No financial compensation | 0.792 | 1.26 |
| Insufficient communication skills | 0.710 | 1.41 |
| Insufficient pharmaceutical knowledge | 0.531 | 1.88 |
| Insufficient clinical medical knowledge | 0.529 | 1.89 |
| Insufficient electronic information skills | 0.612 | 1.64 |
| Not actively introducing to patients | 0.809 | 1.24 |
| Electronic management system barriers | 0.608 | 1.64 |
| Insufficient pharmaceutical workers | 0.641 | 1.56 |
| Lack of regulations in medical institution | 0.675 | 1.48 |
| Lack of dedicated place | 0.573 | 1.75 |
| Lack dedicated time | 0.560 | 1.79 |
| Lack of electronic information system | 0.591 | 1.69 |
| Non-standardized services and documents | 0.601 | 1.66 |
| Self-identified as a non-health care provider | 0.813 | 1.23 |
| Lack of communication with doctors | 0.416 | 2.41 |
| Lack of communication with other health care providers | 0.409 | 2.44 |
| Lack of communication with patients | 0.543 | 1.84 |
| Unable to get medical information | 0.800 | 1.25 |
| Unable to modify the patient's treatment plan | 0.754 | 1.33 |
| Lack of opportunities for further education | 0.619 | 1.62 |
| Lack of time for further education | 0.638 | 1.57 |
| Lack of leadership support from medical institutions | 0.489 | 2.04 |
| Lack of department leadership support | 0.521 | 1.92 |
| Lack of legal and institutional support | 0.674 | 1.48 |

^e^VIF = Variance inflation factor.

**eTable 6**. The questionnaire development

| Steps | Study content |
| --- | --- |
| Step 1 | Access to items: Literature research resulting in a pool of 100 items. |
| Step 2 | Define the dimensions: Interview a total of 10 experts in the field of hospital pharmacy and assign all the above items to five dimensions to work out the Round 1 expert consultation form. |
| Step 3 | The first round of expert consultation: A total of 10 experts in the field of hospital pharmacy were invited, distribute the consultation form by email to the experts and ask them to rate the importance of each item on the 5-point Likert scale (5 points for “very important” and 1 point for “very unimportant”). |
| Step 4 | The first round of revisions: Remove unimportant items to develop the first draft of the Pharmaceutical Care Barriers Questionnaire. |
| Step 5 | The second round of expert consultation: A total of 15 experts in the field of hospital pharmacy were invited, who received emails containing the first draft of the questionnaire and were asked to make a comprehensive evaluation in terms of the rationality of the items under each dimension, and the necessity of adding or deleting any items. |
| Step 6 | The second round of revisions: Refine the questionnaire based on experts' comments and finalize the questionnaire initially. |
| Step 7 | The formation of the final questionnaire: A small sample of 10 postgraduate students with a pharmacy background were randomly included in the test. Based on the results of the test, the questions that were unclear, incomprehensible, ambiguous and questionable were modified and formed into the final questionnaire. |
